# Lithium isotopes differentially modify mitochondrial amorphous calcium phosphate cluster size distribution and calcium capacity

**DOI:** 10.3389/fphys.2023.1200119

**Published:** 2023-09-15

**Authors:** Marshall L. Deline, Joshua Straub, Manisha Patel, Pratigya Subba, Martin Grashei, Frits H. A. van Heijster, Philip Pirkwieser, Veronika Somoza, James D. Livingstone, Michael Beazely, Brian Kendall, Michel J. P. Gingras, Zoya Leonenko, Carmen Höschen, Gertraud Harrington, Katharina Kuellmer, Wangqing Bian, Franz Schilling, Matthew P. A. Fisher, Matthew E. Helgeson, Tobias Fromme

**Affiliations:** ^1^ Chair of Molecular Nutritional Medicine, TUM School of Life Sciences, Technical University of Munich, Freising, Germany; ^2^ Department of Physics, University of California, Santa Barbara, CA, United States; ^3^ Department of Nuclear Medicine, TUM School of Medicine, Technical University of Munich, Munich, Germany; ^4^ Leibniz Institute for Food Systems Biology at the Technical University of Munich, Freising, Germany; ^5^ Chair of Nutritional Systems Biology, TUM School of Life Sciences, Technical University of Munich, Munich, Germany; ^6^ School of Pharmacy, University of Waterloo, Waterloo, ON, Canada; ^7^ Department of Earth and Environmental Sciences, University of Waterloo, Waterloo, ON, Canada; ^8^ Department of Physics and Astronomy, University of Waterloo, Waterloo, ON, Canada; ^9^ CIFAR, MaRS Centre, Toronto, ON, Canada; ^10^ Department of Biology, University of Waterloo, Waterloo, ON, Canada; ^11^ Chair of Soil Science, TUM School of Life Sciences, Technical University of Munich, Munich, Germany; ^12^ Department of Chemical Engineering, University of California, Santa Barbara, CA, United States; ^13^ EKFZ—Else Kröner-Fresenius Center for Nutritional Medicine, Technical University of Munich, Freising, Germany

**Keywords:** mitochondria, calcium, mitochondrial calcium, lithium, lithium bioactivity, amorphous calcium phosphate, isotope distribution

## Abstract

Lithium is commonly prescribed as a mood stabilizer in a variety of mental health conditions, yet its molecular mode of action is incompletely understood. Many cellular events associated with lithium appear tied to mitochondrial function. Further, recent evidence suggests that lithium bioactivities are isotope specific. Here we focus on lithium effects related to mitochondrial calcium handling. Lithium protected against calcium-induced permeability transition and decreased the calcium capacity of liver mitochondria at a clinically relevant concentration. In contrast, brain mitochondrial calcium capacity was increased by lithium. Surprisingly, ^7^Li acted more potently than ^6^Li on calcium capacity, yet ^6^Li was more effective at delaying permeability transition. The size distribution of amorphous calcium phosphate colloids formed *in vitro* was differentially affected by lithium isotopes, providing a mechanistic basis for the observed isotope specific effects on mitochondrial calcium handling. This work highlights a need to better understand how mitochondrial calcium stores are structurally regulated and provides key considerations for future formulations of lithium-based therapeutics.

## 1 Introduction

Lithium is clinically prescribed as a mood stabilizer for a variety of mental health conditions including bipolar disorder, mania and treatment-refractory depression (Gelenberg et al., 1989; Machado-Vieira et al., 2009; Volkmann et al., 2020). Administered in the form of carbonate or citrate salt in solid or liquid formulations, lithium treatment is usually continued over extended periods with the dosage adjusted to maintain a trough level concentration of around 1 mM for therapeutic efficacy and avoidance of adverse effects (Reddy and Reddy, 2014).

Despite its apparent simplicity as a pure elemental ion, the molecular mode of action of lithium has remained largely elusive. Molecular determinants of importance have been argued to include inositol monophosphatase, glycogen synthase kinase-3, binding of nucleotides in competition and/or cooperation with other metal ions, and a plethora of secondary targets (Kerr et al., 2018). In addition to extensively studied individual molecular targets, many cellular events found to be initiated by lithium appear tied to mitochondrial function, among them are: altered rates of reactive oxygen species generation, elimination, and damage accumulation (Shao et al., 2005; Tan et al., 2012), regulators of mitochondrial biogenesis (Castillo-Quan et al., 2016), mitochondrial apoptotic signaling (Aminzadeh et al., 2014), and an enhancement of oxidative phosphorylation (Maurer et al., 2009; Osete et al., 2021). Indeed, lithium is a potent modulator of calcium turnover in brain mitochondria (Shalbuyeva et al., 2007), a central signaling hub with enormous explanatory potential for lithium effects on mitochondria, neuronal function, and consequently on therapeutic outcome. There is a clear need to untangle the many bioactivities of lithium. Here, we specifically focus on the largely unexplored area of lithium modifying mitochondrial calcium storage.

Beyond acting as the hallmark energy converter of the cell, mitochondria are distinct regulators of intracellular calcium, an omnipresent second messenger in a multitude of signaling pathways including synaptic neurotransmitter release (Rossi et al., 2019; Bancroft and Srinivasan, 2022; Bernardi et al., 2022). Mitochondria rapidly sequester massive amounts of intracellular calcium through the internal formation of osmotically inactive amorphous calcium phosphate (ACP) (Wolf et al., 2017; Spät and Szanda, 2018; Petersen et al., 2021). This non-crystalline form of calcium phosphate is thought to consist of 1 nm spheres of the composition Ca_9_(PO_4_)_6_, termed “Posner clusters” (Thomas and Greenawalt, 1968; Posner and Betts, 1975; Wolf et al., 2017). The capacity of calcium sequestration and resistance to calcium overload-induced mitochondrial permeability transition (MPT), which destabilizes ACP and is subject to several modifying parameters including the availability of substrate and oxygen, membrane potential, and nucleotide concentrations, specifically of adenosine triphosphate which is believed to stabilize ACP (Lehninger, 1970; Becker, 1980; Chalmers and Nicholls, 2003; Deline et al., 2021). Metal ion concentrations constitute a further source of altered Posner cluster stability (Blumenthal et al., 1977; Silver and Sordahl, 1980; Shalbuyeva et al., 2007), since theoretical calculations of alternative cluster configurations show that substitution of metal ions for the central calcium ion modifies the energetic favorability of the Posner cluster complex (Swift et al., 2018). Intriguingly, the central calcium of a Posner cluster can be replaced by two lithium ions to form a stabilized complex (Swift et al., 2018), a mechanism potentially underlying lithium-mediated changes in brain mitochondrial calcium capacity (Shalbuyeva et al., 2007).

Lithium naturally occurs as two isotopes, lithium-7 (^7^Li, 92.5% natural abundance) and lithium-6 (^6^Li, 7.5% natural abundance) that differentially affect animal behavior (Sechzer et al., 1986; Ettenberg et al., 2020). Here, we corroborate the modulation of mitochondrial calcium handling by lithium, uncover a differential impact of lithium isotopes in an organ specific manner, and report on an exhaustive approach probing the potential mechanisms behind disparities of isotopic lithium.

## 2 Materials and methods

### 2.1 Mitochondrial isolation

Liver mitochondria were isolated from C57BL6/N mice through differential centrifugation as previously described (Deline et al., 2021). Brain mitochondria were isolated in a similar manner with the following additional steps to reduce synaptosome contamination. The homogenate of three brains was centrifuged at 1,300 relative centrifugal force (RCF) for 3 min at 4°C. Half of the supernatant was collected into a new tube, while the remainder was used to resuspend the pellet which then underwent another round of homogenization. The second homogenate was combined with the collected supernatant and centrifuged again at 1,300 RCF for 3 min. The resulting supernatant was then centrifuged at 12,000 RCF for 10 min and the mitochondria and synaptosome fraction was brushed away from the erythrocyte pellet, transferred to a fresh tube, resuspended in isolation buffer, and centrifuged again at 1,200 RCF for 10 min. The pelleted non-synaptic mitochondria and synaptosomes were resuspended in 1 mL of isolation buffer and added to isolation buffer containing 13% OptiPrep (Serumwerk) which was then layered on top of isolation buffer containing 23% OptiPrep and centrifuged at 30,700 RCF for 10 min to form a pellet of free mitochondria and a layer of synaptosomes at the interface of the discontinuous gradient. Non-synaptic mitochondria were resuspended in isolation buffer lacking EGTA and BSA and centrifuged at 16,700 RCF for 10 min. The final pellet was resuspended in isolation buffer lacking EGTA and BSA, and protein content was determined by the Biuret method.

### 2.2 Mitochondrial permeability transition assay

The onset of MPT was determined with a TECAN Infinite M200 microplate reader (TECAN grp.) following fluctuations in the fluorescence emitted by the extramitochondrial calcium sensor, Calcium Green-5N (ThermoFisher), as previously described (Deline et al., 2021). Mitochondria (0.2 mg/mL) were suspended in Calcium Assay Buffer [10 mM HEPES, 2 mM KH_2_PO_4_, 1 µM rotenone, 2 mM succinate, 1 mM MgCl_2_, 2.5 µM oligomycin, and 0.4 µM Calcium Green-5N, at pH 7.0] containing either 125 mM KCl or 125 mM LiCl, as indicated. Trace calcium was removed from buffers using Chelex-100 resin (Sigma) regenerated to the potassium form. Fluorescence of the calcium sensor was determined once per minute for at least 1 hour after calcium (350 nmol/mg mitochondrial protein) was applied. Onset of MPT was then defined as the maximal rate change in fluorescence after completion of mitochondrial calcium uptake.

### 2.3 Mitochondrial calcium accumulation and efflux assays

Mitochondrial calcium accumulation was determined with a TECAN Infinite M200 microplate reader (TECAN grp.) in a similar manner as carried out in the above MPT onset determinations. Mitochondria (0.2 mg/mL) were buffered by Calcium Assay Buffer supplemented with 125 mM KCl, 125 mM ^6^LiCl, 125 mM ^7^LiCl, 1 mM ^6^LiCl (in 124 mM KCl), or 1 mM ^7^LiCl (in 124 mM KCl) and subjected to multiple additions of 125 nmol CaCl_2_/mg protein once every 10 min for 100 min. The amount of calcium sequestered by mitochondria was calculated as the difference between the fluorescence signal corresponding to the injection of 125 nmol CaCl_2_ and the minimum fluorescence which followed. Calcium accumulations from all injections in the series were then summed to determine the maximal accumulation. To determine calcium efflux rates, mitochondria were loaded with 125 nmol CaCl_2_/mg protein for 10 min as above and then treated with 0.5 µM of the mitochondrial calcium uniporter (MCU) inhibitor, ruthenium red. The fluorescence increase of Calcium Green-5N over a 40 s time window following ruthenium red treatment was used to calculate calcium efflux rates.

### 2.4 Determination of mitochondrial membrane potential effects

The membrane potential of mitochondria buffered by 10 mM HEPES supplemented with 1 µM rotenone, 2.5 µM oligomycin, and either 125 mM KCl, 125 mM ^6^LiCl, 125 mM ^7^LiCl, 1 mM ^6^LiCl (in 124 mM KCl), or 1 mM ^7^LiCl (in 124 mM KCl) were assessed by fluorescence of 2 µM Safranin O on a TECAN microplate reader, measuring once every min. After 10 min, a membrane potential was generated by the addition of 2 mM succinate and 1 mM ADP. The contribution of the membrane potential to the fluorescence change was confirmed by applying 2.5 µM of the complex III inhibitor, antimycin A. The fluorescence signal was normalized to the first 3 min of baseline signal. After 5 min of succinate and ADP addition, a 5 min window of Safranin O signal was averaged and compared by one-way ANOVA.

### 2.5 Determination of lithium distribution across mitochondrial and synaptic membranes

Mitochondria isolated from mouse liver and synaptosomes were suspended in a 125 mM buffer containing 62.5 mM ^6^LiCl (95% isotopic purity, Sigma Aldrich) and 62.5 mM ^6^LiCl (99% isotopic purity, Sigma Aldrich) at 2 mg/mL and stirred at 37°C. Mitochondrial samples were energized by adding 2 µM rotenone and 2 mM succinate. 1 mL samples were taken and centrifuged for 3 min at 16,000 RCF and 4°C. The supernatant and pellet were collected separately. The pellet was dissolved in 100 µL of 5% sodium deoxycholate and passed through a 0.2 µm filter. Isotope ratio measurements and a screening of trace-level contaminations in Li stock solutions were conducted using a Nexion 5000 ICP-MS system (PerkinElmer). Regarding isotope ratios, Li stock solutions from certified materials, including a natural abundance reference, as well as the previously prepared samples were diluted in 2% HNO_3_ (ROTIPURAN®Supra 69%, Carl Roth). To achieve a high isotope ratio precision, collisional focusing was applied by pressurizing the dynamic reaction cell with 0.5 mL/min helium. Other method parameters were as follows: MS/MS scan mode, focusing ion guide mode, 1000 sweeps/reading, 10 readings/replicate and 10 replicates, with dwell times of 1 ms for ^7^Li and 2 ms for ^6^Li. Possible trace level contaminations of Li stock solutions were examined after calibration with Instrumental Calibration Standard 2 and Alternate Metals solution (PerkinElmer), with Scandium (PurePlus, PerkinElmer), Rhodium (Certipur^®^, Supelco) and Rhenium (Aristar^®^ VWR) as internal standards.

### 2.6 Lithium isotope fractionation across the HT22 neuronal cell plasma membrane using ICP-MS

#### 2.6.1 HT22 cell culture and Li treatment

HT22 cells were cultured in full growth media [DMEM and HAM’s F12 (1:1) (Fisher #SH20361), 10% fetal bovine serum, 100 U/mL penicillin and 100 μg/mL streptomycin] at 37°C, 5% CO_2_ and passaged every 48 h using 0.25% trypsin/0.1% EDTA in a 1:10 dilution. HT22 cells were plated at 80,000 cells/mL in 6-well plates (2 mL each well), and grown in full growth media at 37°C, 5% CO_2_ for 24 h to 60% confluency, then differentiated with neurobasal media (supplemented with 5 mM N_2_ supplement and 5 mM L-glutamine) for 24 h. After differentiation, the plates were treated with lithium isotopes in the form of lithium carbonate (obtained from Sigma) for 24 h. The ^6^Li-dominant salt contained approximately 95% ^6^Li and 5% ^7^Li. The ^7^Li-dominant salt (natural) contained approximately 92% ^7^Li and 8% ^7^Li. A third treatment group treated with a 50/50 mixture of ^6^Li-dominant salt and ^7^Li-dominant salt solutions was also analyzed. After 24 h treatment at 4 mM and 8 mM lithium, the cells were washed twice with 1 mL of phosphate-buffered saline (PBS) solution, then 70 µL of lysis buffer was added [20 mM Tris-HCl pH 7.5, 150 mM NaCl, 1 mM Ethylenediaminetetraacetic acid (EDTA), 1 mM ethylene glycol tetraacetic acid (EGTA), 30 mM sodium pyrophosphate, 1 mM betaglycerophosphate, 1 mM sodium orthovanadate (Na_3_VO_4_), and 1% triton]. These protocols were adapted from protein-harvesting techniques often used for Western blotting. The cells were scraped off the plate and homogenized using fine-gauge syringe needles. After centrifugation at 14,000 RCF at 4°C for 20 min, the supernatant was obtained and frozen at −20°C until analyzed. A sample of the media, the PBS wash, and the cell lysate for each well was collected and analyzed by ICP-MS.

#### 2.6.2 HT22 ICP-MS methodology

We developed protocols for measurement of Li isotope abundances in biological material using triple quadrupole inductively coupled plasma mass spectrometry (QQQ-ICP-MS). Specifically, each cell sample was digested in a mixture of 0.5 mL concentrated (67%–70%, Fisher Scientific trace metal grade) HNO_3_ and 0.5 mL 30% H_2_O_2_ (Millipore Suprapur) for 1 h at 110°C to destroy organic matter, then evaporated to dryness. Each sample was then diluted in 3 mL of 2% trace metal grade HNO_3_ for analysis. The concentrations of ^6^Li and ^7^Li were determined on an Agilent 8800 QQQ-ICP-MS operated in MS/MS mode using a hot plasma (1550 W) and with helium as a collision cell gas (2.5 mL/min). Tuning was carried out with a 1 ppb multi-element solution, and with minimization of cerium oxide and Ce^2+^ formation (156/140 and 70/140 ratios ≤ 2%). Each sample solution measurement comprised 10 replicate analyses, with 1000 sweeps of the mass spectrum and an integration time of 2 seconds for each lithium isotope (dwell time of 2 ms for each Li isotope) per replicate analysis. Sample uptake and stabilization times were 60 s and 30 s, respectively. Between samples, a rinse sequence was performed that first comprised 15 s ultrapure water and was followed by three 60 s rinses in separate bottles of 2% trace-metal grade HNO_3_. Scandium was used as an internal element standard to correct for instrument drift. Detection limits and background equivalent concentrations were less than 0.01 ppb for both lithium isotopes. Instrumental accuracy was verified using multiple United States Geological Survey (USGS) water standards, and using check standards comprising ^7^Li-rich (natural), ^6^Li-rich, and a 50/50 mix of ^6^Li-dominant and ^7^Li-dominant solutions.

### 2.7 NanoSIMS determination of spatial distribution of lithium isotopes within NIH/3T3 cells

To determine the spatial distribution of the lithium isotopes at the microscale using NanoSIMS, NIH/3T3 cells were grown in high glucose DMEM (D5796, Sigma) on silicon wafers and exposed to 400 µM of LiCl for 48 h. After the wafer was dipped in PBS to wash away media, the PBS was quickly wicked away with a tissue and the cells were cryo-frozen in 2-methylbutane cooled in a liquid nitrogen bath. To compare the lithium incorporation with the original source, a sample of the media was prepared by dropping it on a silicon wafer and allowing it to dry. After inspection with a reflectance light microscope (Zeiss Axio Imager Z2) to detect the cells and media distribution on the wafer, the samples were coated with a conductive Au/Pd layer (ca. 30 nm, Polaron Emitech SC7640 sputter coater) to account for charging effects under the NanoSIMS ion beam. Prior to the NanoSIMS measurement, contaminants and the Au/Pd coating layer were locally sputtered away using a high primary beam current (pre-sputtering/implantation) until the secondary ion emission reached a steady state. The O^**−**^ primary ion beam of the RF plasma source with an impact energy of 16 keV (ca. 10 pA) was locally scanned over the sample to record the spatial distribution of the lithium secondary ions ^6^Li^+^ and ^7^Li^+^ produced by sputtering. The secondary ions were detected on electron multipliers with an electronic dead time fixed at 44 ns. Using a dwell time of 1 ms/pixel, 256 × 256 pixels for a 20 μm × 20 μm field of view, 60 planes for the cells and 30 planes for the media were collected. NanoSIMS images were analyzed with Open-MIMS image plugin (Gormanns et al., 2012). The measurements were corrected for dead time and drift and 10 or 20 planes were accumulated into a single frame providing three frames for each cellular region of interest (ROI) in the cell medium and intracellular samples, respectively. Isotopic ratio was calculated pixel by pixel and the data were used to spatially identify the ratio of lithium secondary ions ^6^Li^+^ and ^7^Li^+^.

### 2.8 Integration of lithium-6 and lithium-7 into amorphous calcium phosphate determined by ^31^P NMR spectroscopy

Solution NMR relaxation experiments were performed using a Bruker Avance NEO 500 MHz spectrometer with a CryoProbe Prodigy BBO probe, using Wilmad-Lab Glass 5 mm Thin Wall Precision NMR tubes. A 45° pulse with a duration of 6.75 microseconds was employed, with 10 s recycle delay, 1 s acquisition time, and 32 total scans. All chemical shifts were referenced to 1 M phosphoric acid located in a coaxial insert.

### 2.9 Lithium-6 and lithium-7 effects on amorphous calcium phosphate stoichiometry determined by ^31^P-NMR spectroscopy

Experiments to probe the calcium-to-phosphate stoichiometry in amorphous calcium phosphate and to investigate changes upon lithium addition due to calcium replacement by lithium ions were performed on an AV 500 MHz NMR Spectrometer (Bruker Biospin) with a cryo-cooled Quattro Nucleus Probe (QNP) head. A pulse-and-acquire 1H-decoupled 1D NMR experiment with repetition time = 10 s, flip angle = 30°, spectral width = 20.2 kHz, 65,536 spectral points with no averaging and no dummy scans to avoid relaxation-related misquantification was acquired on amorphous calcium phosphate produced in a bulk methodology adapted from a previously published study (Zhang et al., 2007). Calcium chloride was added to a solution of 20 mM Na_2_HPO_4_, maintained at pH 10 with aqueous ammonia, to achieve varying Ca:PO_4_ ratios. The tubes were inverted to mix and calcium phosphate precipitates were allowed to settle at room temperature for 5 min. Subsequently, the precipitates were mixed with D_2_O and transferred to thin walled glass NMR tubes for data acquisition. The spectra were line-broadened by 1 Hz, phased and baseline-corrected prior to quantification. As phosphate bound in amorphous calcium phosphate exhibits extremely broad lines (Supplementary Figure S5) relative to inorganic phosphate, its signal is not detectable for a single transient as applicable for this experiment. Therefore, the phosphate peak only accounts for the free, unbound phosphate in solution which should disappear as soon as all phosphate is bound in amorphous calcium phosphate. Peak integrals of inorganic phosphate were determined with MNova software (Mestrelab Research S. L.). Experiments for control samples and samples containing 150 mM ^6^Li or 150 mM ^7^Li were performed identically.

### 2.10 Dynamic light scattering

Dynamic light scattering (DLS) was conducted with a BI-200SM Goniometer System with a TurboCorr correlator (Brookhaven Instruments) and a Cobolt Samba 500 mW laser at 532 nm (HÜBNER Photonics). DLS measurements were carried out at a 90° scattering angle at 37°C with 200 correlation channels ranging from 100 ns to 100 ms and sampling rates of 100 ns, 5 µs, and 50 µs, depending on the channel delay. Experiments were conducted by preparing mother solutions of 125 mM lithium chloride and 2 mM sodium phosphate, then buffering to the desired pH with 0.2 M NaOH. These solutions were filtered using 0.2 μm cellulose acetate filters and monitored for 120 s with DLS to confirm solution cleanliness, prior to calcium addition. For each pH and lithium isotope, ten aliquots were then prepared. For each aliquot, calcium was added followed by rapid vortex mixing of the solution for 5 s. DLS acquisition was begun after 5 s to allow for any internal flows to subside. The DLS signal was then measured in 10 s increments over 300 s. For reported particle sizes, the method of cumulants was used with a quadratic fit to extract a mean diameter. An ANOVA with a repeated measures in the time factor was performed to assess the significance of the data.

### 2.11 Statistical analyses

All statistical analyses and linear regression calculations were performed with the Prism 9 software (GraphPad).

## 3 Results

### 3.1 Lithium-6 delays mitochondrial permeability transition more than lithium-7

**FIGURE 1:**
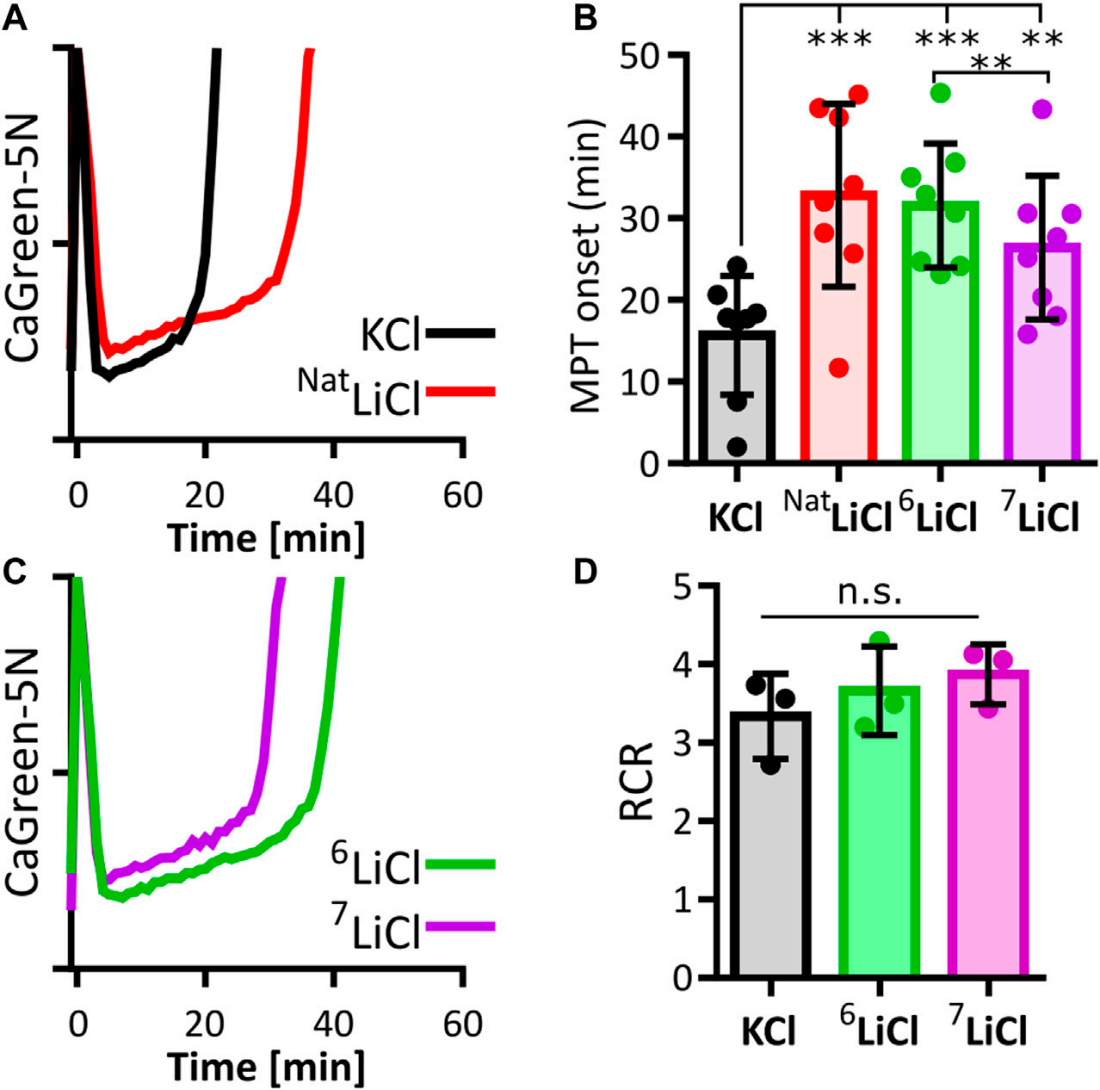
Lithium-6 delays mitochondrial permeability transition longer than lithium-7. Onset of mitochondrial permeability transition (MPT) in succinate respiring liver mitochondria was compared between potassium (KCl), natural lithium chloride (^Nat^LiCl, consisting of ∼92.5% ^7^Li and ∼7.5% ^6^Li), lithium-6 (^6^LiCl) and lithium-7 chloride (^7^LiCl). **(A)** Representative traces of extramitochondrial calcium (CalciumGreen-5N) for KCl (black) and ^Nat^LiCl (red) exposed mitochondria supplemented with 350 nmol/mg calcium at time point zero. **(B)** MPT onset times are compared. Each biological replicate (*n* = 8) is represented as the mean of 6–12 technical replicates. All lithium treatments significantly delay MPT compared to KCl. ^6^LiCl significantly delays MPT compared to ^7^LiCl as determined by ANOVA followed by post hoc Tukey’s multiple comparisons test, ** = *p* < 0.005, *** = *p* < 0.001. **(C)** Representative traces of ^6^LiCl (green) and ^7^LiCl (magenta) exposed mitochondria supplemented with 350 nmol/mg calcium at time point zero. **(D)** Lithium does not alter mitochondrial respiration efficiency. Average respiratory control ratio (RCR) of liver mitochondria from 3 biological replicates in the presence of 125 mM KCl, ^6^LiCl, or ^7^LiCl. No statistical difference was found when assessed by ANOVA.

Mitochondrial calcium overload leads to the opening of unspecific pores in the inner mitochondrial membrane, a process called mitochondrial permeability transition (MPT), which melts the ACP pool and triggers mitochondrial calcium efflux (Halestrap, 1999; Kokoszka et al., 2004; Bernardi et al., 2022). Lithium-induced stabilization of mitochondrial ACP could therefore prevent or delay the onset of this terminal event. Indeed, MPT onset of succinate respiring mouse liver mitochondria exposed to 125 mM lithium chloride in its natural isotope ratio consistently occurred later than in the presence of potassium chloride (Figures 1A, B), in agreement with an earlier observation and the predicted structure of lithium-ACP interaction (Shalbuyeva et al., 2007; Swift et al., 2018).

The two naturally occurring isotopes, ^6^Li and ^7^Li, have been reported to elicit distinct effects on mammalian behavior. ^6^Li, but not ^7^Li, abrogates certain manic effects triggered by ketamine in mice, and promotes certain maternal behaviors in female rats (Sechzer et al., 1986; Ettenberg et al., 2020). These observations prompted us to expand our investigation to include a search for lithium isotope-specific effects on MPT onset. In liver mitochondria exposed to ^6^Li chloride, calcium induced MPT reproducibly occurred later compared to an equal concentration of ^7^Li chloride (Figures 1B, C). An impact of either lithium isotope on mitochondrial respiration was ruled out as a contributing factor by respirometry (Figure 1D). These data therefore indicate that the influence of lithium on liver mitochondrial calcium uptake, capacity and/or stability is dependent on isotopic identity.

### 3.2 Lithium influences mitochondrial calcium accumulation in an isotopic and tissue dependent manner

**FIGURE 2:**
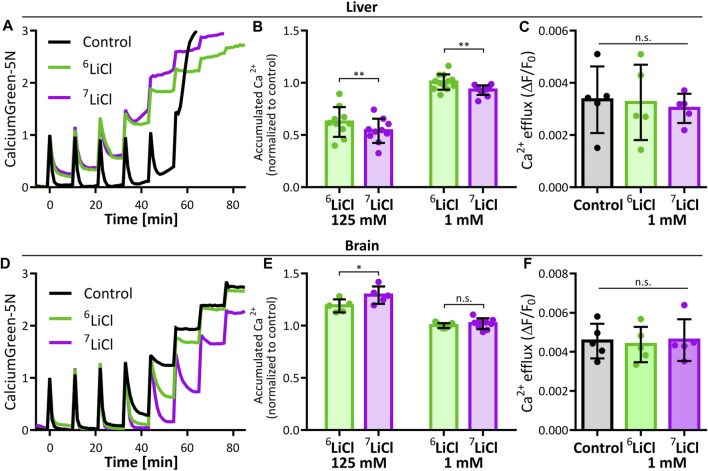
Lithium-7 is a potent effector of mitochondrial calcium accumulation. The effect of lithium isotopes on liver and brain mitochondria maximal calcium accumulation were compared to potassium (Control). **(A)** Representative traces of 125 mM KCl (Control, black), ^6^LiCl (green), or ^7^LiCl (magenta) treated mitochondria given multiple additions of 125 nmol Ca/mg starting at time point zero. **(B)** Maximal calcium accumulation of 125 mM or 1 mM lithium isotope treated liver mitochondria normalized to control. Biological replicates (125 mM, *n* = 10 and 1 mM, *n* = 12) are plotted as the means of 2-4 technical replicates. Significance was determined by ANOVA with *post hoc* Sidak’s multiple comparisons test. **(C)** Calcium efflux rate of liver mitochondria in the presence of 1 mM ^6^LiCl, ^7^LiCl, or KCl (Control) are compared. Rates were calculated by making linear regressions of traces following 0.5 µM ruthenium red treatment of 125 nmol Ca/mg loaded mitochondria. No significant difference was detected by ANOVA, *n* = 5 biological replicates. **(D)** Representative traces of brain mitochondria in the presence of by 125 mM KCl (Control, black), ^6^LiCl (green), or ^7^LiCl (magenta) and given multiple additions of 125 nmol Ca/mg starting at time point zero. **(E)** Maximal calcium accumulation of 125 mM or 1 mM lithium isotope treated brain mitochondria normalized to control. Biological replicates (125 mM, *n* = 5 and 1 mM, *n* = 9) are plotted as the means of 2-4 technical replicates. Significance was determined by ANOVA with post hoc Sidak’s multiple comparisons test. **(F)** Calcium efflux rate of brain mitochondria in the presence of 1 mM ^6^LiCl, ^7^LiCl, or KCl control are compared. Rates were calculated as in **(C)**. No significant difference was detected by ANOVA, *n* = 5 biological replicates. For all, bar height represents the mean of replicates and error bars the standard deviation, * *p* < 0.05, ** *p* < 0.005.

We sought to further understand the isotope dependence of lithium chloride delaying the onset of calcium-induced MPT. Calcium acts within the mitochondrial matrix to induce MPT as a function of calcium amount. We therefore determined if calcium uptake is susceptible to lithium isotope effects on liver mitochondria. Calcium Green-5N fluorescence was used to determine maximal calcium accumulation by summing the total calcium removed from the buffer by mitochondria suspended in 125 mM ^6^LiCl or ^7^LiCl and normalized to the 125 mM KCl control. The presence of lithium interfered with the accumulation of calcium by liver mitochondria (Figures 2A, B), suggesting that the overall lithium-mediated delay in MPT onset, demonstrated in Figures 1A, B, is at least in part due to a reduction in mitochondrial matrix calcium abundance. Interestingly, the ^7^Li isotope treatment displayed a more potent interference with calcium accumulation compared to ^6^Li treatment (Figure 2B). Importantly, the differential effect of lithium isotopes on calcium accumulation persisted down to the lowest lithium concentration tested, 1 mM, which fits within the range reported for lithium content in the serum and intracellular space (0.5–1 mM) resulting from lithium treatment (Figure 2B) (Gelenberg et al., 1989; Kabakov et al., 1998; Volkmann et al., 2020).

The duration between calcium uptake and MPT onset is expected to negatively correlate with the amount of accumulated calcium if it is linked by causality, e.g., a higher calcium uptake should lead to a quicker MPT onset and *vice versa*. Counter to this assumption, we found that mitochondria treated with 125 mM ^6^Li accumulate more calcium than those treated with 125 mM ^7^Li, and delays MPT onset longer than the 125 mM ^7^Li treatment. This divergence led us to explore if other factors of mitochondrial calcium handling or differences in trace ions of the salt stocks explain these isotope effects. Calcium concentration in the assay medium is a function of both mitochondrial calcium uptake and release. We excluded a lithium isotope effect on mitochondrial calcium export, at a concentration of 1 mM lithium, by measuring calcium release rates in the presence of the MCU inhibitor, ruthenium red, following a pre-loading of mitochondria with 125 nmol CaCl_2_/mg (Figure 2C). Further, we carefully controlled potential secondary effects of contaminating ions in our pure lithium isotope stocks. An analysis by inductively coupled plasma mass spectrometry (ICP-MS) identified marginally higher trace ion levels in the ^6^LiCl stock (Mg^2+^, Na^+^, Cu^2+^) compared to the ^7^LiCl stock. Mimicking the trace ion content of the ^6^LiCl in the ^7^LiCl stock had no impact on calcium accumulation of mitochondria treated at the 125 mM lithium chloride concentration (Supplementary Figure S1). We conclude that ^6^Li itself caused both an increased mitochondrial calcium accumulation and a longer delay of MPT onset compared to ^7^Li treatment.

Lithium is clinically applied to elicit psychological and behavioral effects. Our findings on clinically relevant lithium concentrations (1 mM) acting on liver mitochondria are thus primarily relevant to the etiology of adverse effects brought on by lithium treatment. We accordingly extended our study to brain mitochondria to assess if similar lithium isotope specific actions are neurologically relevant. Due to the much larger calcium capacity of brain mitochondria (Chalmers and Nicholls, 2003; Serna et al., 2022), MPT cannot be induced under the conditions performed in Figure 1 (Supplementary Figure S2). This tissue-specificity of mitochondrial calcium capacity and tolerance against MPT is well known (Chalmers and Nicholls, 2003; Serna et al., 2022). We thus repeated the experiment of stepwise calcium addition to mitochondria isolated from murine brain tissue to assess maximal calcium accumulation. Strikingly, and in stark contrast to liver mitochondria, 125 mM lithium chloride enhanced maximal calcium accumulation of brain mitochondria, and ^7^Li was the more potent enhancer compared to ^6^Li (Figures 2D, E). At a concentration of 1 mM lithium, brain mitochondrial calcium accumulation and efflux rates were not sensitive to lithium isotopes (Figures 2E, F). Significance of the calcium accumulation results were determined by a two-way ANOVA comparing biological replicates of the 125 mM and 1 mM lithium treatments. Calcium uptake in the presence of potassium or lithium could be fully inhibited by ruthenium red, in line with an uptake mechanism dependent on the ruthenium red sensitive mitochondrial calcium uniporter (Supplementary Figures S3A, B). Further, the resting membrane potential of neither liver nor brain mitochondria were affected by any of the lithium treatments tested (Supplementary Figure S3C–F).

Lithium interacting with calcium processing in a concentration- and isotope-specific manner, as shown here in both liver and brain mitochondria, provides a new mechanistic perspective on the bioactivity of lithium.

### 3.3 Lithium isotopes are not differentially distributed across cellular membranes

The two lithium isotopes differentially altered mitochondrial calcium management. In principle, this phenomenon can be caused by either of two underlying mechanisms: First, lithium isotopes may differentially compartmentalize within cells, e.g., through different transport/diffusion kinetics across membranes and thus be available to transporters as counter-ions in different concentrations. Second, lithium isotopes may directly alter ACP within the mitochondrial matrix, e.g., as an alternative component of the ACP subunit, as predicted earlier for the Posner cluster (Swift et al., 2018).

**FIGURE 3:**
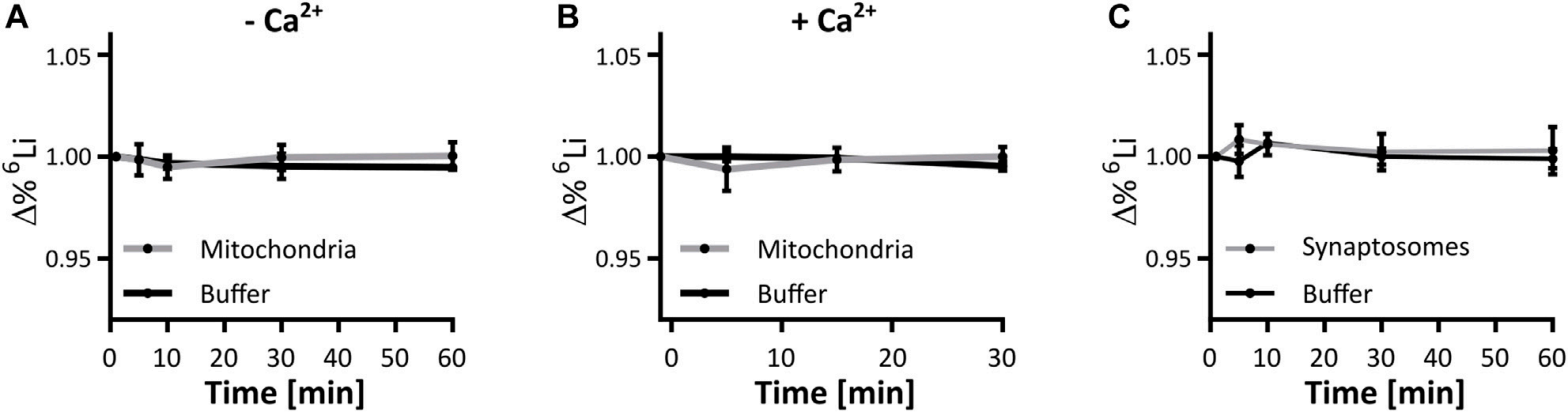
Lithium isotope specific distribution is unhindered by mitochondrial or synaptic membranes. Mitochondrial and synaptic membranes were tested for their ability to segregate lithium isotopes when suspended in a buffer containing 125 mM. LiCl (45:55, ^6^Li:^7^Li). **(A)** Change in the ^6^Li/^7^Li ratio over time in respiring liver mitochondria and the supporting buffer, compared to the initial time point, *n* = 3. **(B)** Change in the ^6^Li/^7^Li ratio over time in respiring liver mitochondria presented with 125 nmol/mg CaCl_2_ for 5 min, and the supporting buffer when compared to the initial time point, *n* = 3. **(C)** Change in the ^6^Li/^7^Li ratio over time in synaptosomes and the supporting buffer, when compared to the initial time point, *n* = 3. No point in **(A–C)** was significantly different when assessed by ANOVA.

We determined lithium isotope distribution across the mitochondrial inner membrane. Mitochondria isolated from mouse liver were exposed to respiration buffer containing 125 mM LiCl of close to equimolar isotope abundance, i.e. 45 % ^6^Li and 55% ^7^Li, verified by ICP-MS. Over the course of an hour, samples of mitochondria and buffer were taken and analyzed by ICP-MS to detect a possible change in isotope ratio in either fraction. At no point did the lithium isotope ratio within mitochondria nor in the buffer deviate from the starting value, suggesting that mitochondrial membranes and their ion transport systems act on both isotopes without selectivity (Figure 3A). This assay was repeated in the presence of 125 nmol CaCl_2_/mg mitochondrial protein, to mimic the mitochondrial calcium handling assays performed above. Again, no change was identified in the ratio of the lithium isotopes (Figure 3B). We conclude that respiring liver mitochondria do not preferentially distribute either lithium isotope across their membranes, neither in the absence, nor in the presence of calcium.

**FIGURE 4:**
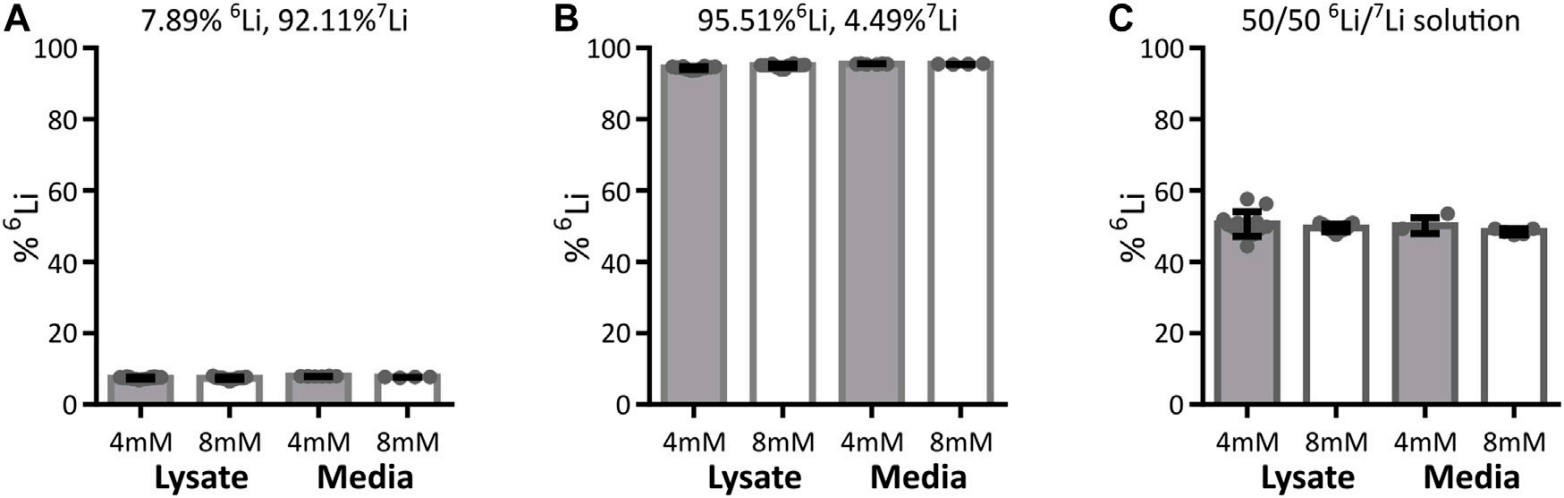
Lithium isotope distribution across HT22 neuronal cell plasma membrane. The detected lithium isotope ratio in the HT22 cells (cell lysates including cellular membrane) and outside HT22 cells (media) after treatment with various concentrations and isotope ratios of ^6^Li and ^7^Li. Data are presented as mean ± standard deviation. Cells were treated with either 4 mM or 8 mM total lithium concentration, with **(A)** natural lithium (92.11% ^7^Li, 7.89% ^6^Li), 4 mM lysate *n* = 18, medium *n* = 8, 8 mM lysate *n* = 15, medium *n* = 4, **(B)** ^6^Li enriched (4.49% ^7^Li, 95.51% ^6^Li), 4 mM lysate *n* = 12, medium *n* = 6, 8 mM lysate *n* = 14, medium *n* = 4, or **(C)** a 50/50 lithium isotope mix (equal parts media described in A and B), 4 mM lysate *n* = 13, medium *n* = 4, 8 mM lysate *n* = 15, medium *n* = 5. Regardless of the treatment conditions, the detected intracellular lithium isotope ratio (lysate) does not show any significant difference from the detected extracellular isotope ratio (media) when assessed by ANOVA.

Within a cell, mitochondria have the possibility to be exposed to different lithium concentrations as a result of the plasma membrane selectively segregating lithium isotopes. We thus expanded the analysis to the neuronal plasma membrane in a model system of murine synaptosomes. As with isolated mitochondria, the lithium isotope ratio was unchanged at any time point during the exposure of synaptosomes to lithium isotopes (Figure 3C). To further confirm this finding, a neuronal-derived cell model (HT22 cells) and ICP-MS analysis were used to evaluate if there is selective passage of lithium isotopes across the neuronal plasma membrane of such cells. The HT22 neuronal cell line is a murine hippocampal-derived cell model that has been used extensively in neurotoxicology and signaling studies to model neuronal cells. The effect of lithium isotopes, provided in the carbonate form, was tested in three groups: first, the ^6^Li-dominant salt contained approximately 95% ^6^Li and 5% ^7^Li; second, the ^7^Li-dominant salt (natural) contained approximately 92% ^7^Li and 8% ^6^Li; and the third group was treated with a 50/50 mixture of ^6^Li-dominant salt and ^7^Li-dominant salt solutions. Cells were treated for 24 h with 4 and 8 mM lithium, concentrations previously shown to penetrate into HT22 cells without toxicity (Livingstone et al., 2023). None of the conditions produced a change in the intracellular lithium isotope ratio from that present in the medium when assayed by ICP-MS and assessed by ANOVA (Figure 4). The form of lithium salt used, chloride (mitochondria and syntaptosomes) or carbonate (HT22 cells), had no effect on the lithium isotope distribution.

**FIGURE 5:**
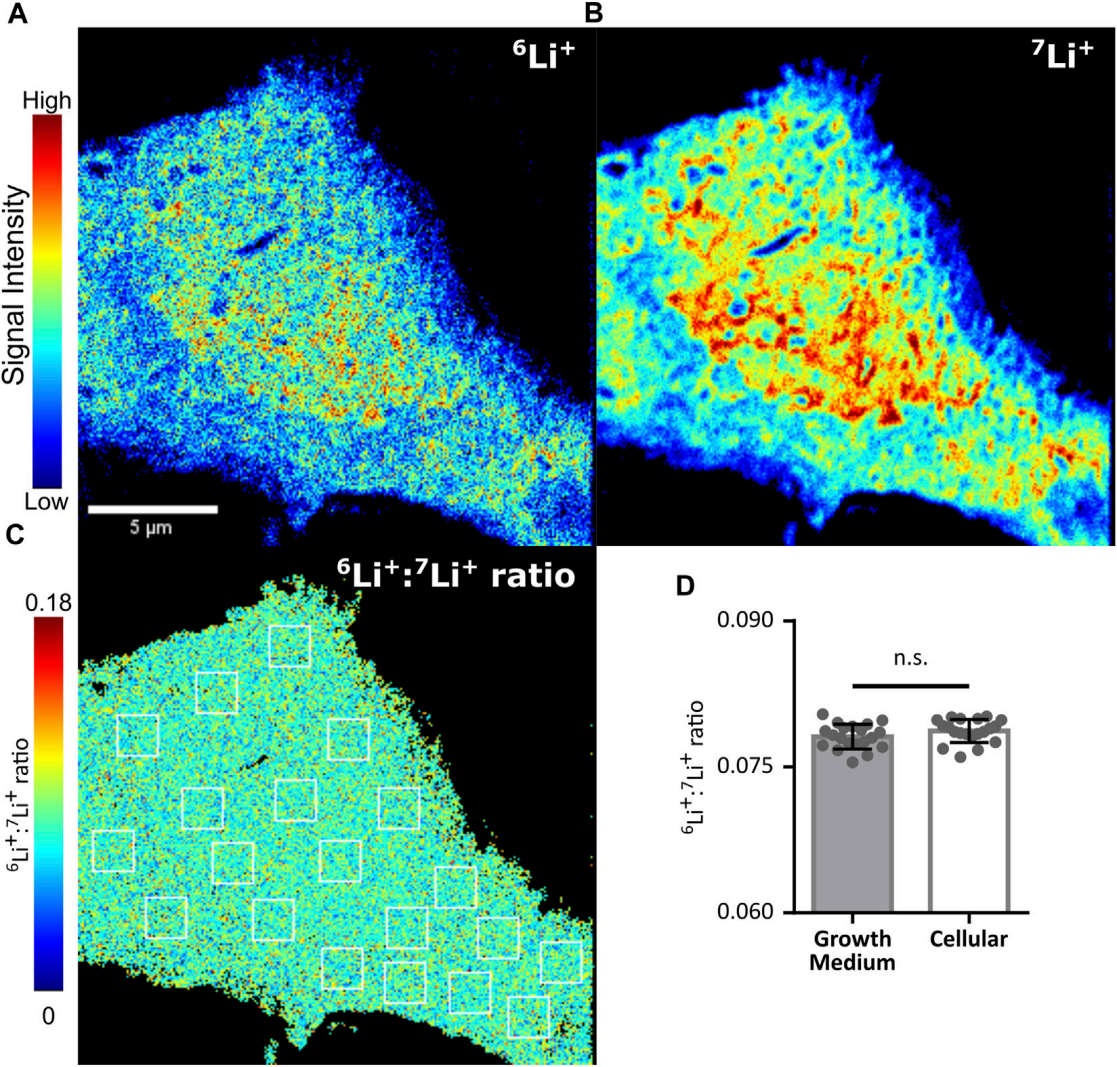
Lithium isotope specific distribution is unhindered by cellular membranes. Lithium isotope distribution throughout an NIH/3T3 cell treated with 400 µM LiCl for 48 h, as determined by NanoSIMS. **(A)** Distribution of lithium-6 (^6^Li^+^). **(B)** Distribution of lithium-7(^7^Li^+^). **(C)** Pixel by pixel determination of the ^6^Li+/^7^Li + ratio from A/B. Twenty 1.56 µm^2^ regions of interest (ROI, white boxes) were drawn within the cell represented and equivalent ROIs were used in a growth medium control to assess subcellular differences in ^6^Li+/^7^Li + ratios. **(D)** Mean values from ROIs taken from C (open bar) or equivalent ROIs from a growth medium control (shaded bar). No difference was identified when assessed by ANOVA. Error bars plotted as standard deviation of the 20 ROIs.

Finally, we sought to identify hot spots within the cell where either lithium isotope may be differentially accumulated by using the NanoSIMS mass spectrometry-based imaging technique. This method provides a means to determine isotopic distribution at high spatial resolution (Nuñez et al., 2018; Bonnin and Rizzoli, 2020; Bonnin et al., 2021). The O^**−**^ primary ion source of the NanoSIMS is capable of distinguishing the localization of ^6^Li and ^7^Li with a spatial resolution of 100 nm, high mass resolution and a high secondary ion yield. NIH/3T3 cells, which have a large lamellar area, were chosen to increase the definable area of potential lithium compartments. Cells grown on a silicon wafer were treated with 400 µM natural LiCl (^7^Li: ∼92.5%, ^6^Li: ∼7.5%) for 48 h prior to cryo-freeze in liquid N_2_ cooled 2-methylbutane and freeze drying. Each of the two lithium isotopes was found to be present in a characteristic pattern representative of lithium sequestration into diverse subcellular structures. However, there was no area within the cell where the ratio of lithium isotopes was altered beyond the precision of the NanoSIMS device as detected from the measurement on the media (mean ^6^Li/^7^Li ratio = 0.079 ± 0.0012), indicating that neither plasma membrane nor internal membranes preferentially sequester one of the lithium isotopes (Figure 5). Such lithium isotope signals were absent in cells treated with 400 µM NaCl as a negative control (Supplementary Figure S4).

Taken altogether, these data quantifying lithium abundance acquired by multiple model systems and an array of state-of-the-art detection techniques lead us to conclude that lithium isotopes distribute similarly throughout a cell. Thus, differential effects imparted by lithium isotopes on mitochondrial calcium handling are not the result of differences in intra-mitochondrial nor intracellular isotopic lithium concentrations.

### 3.4 Lithium integrates into amorphous calcium phosphate

**FIGURE 6:**
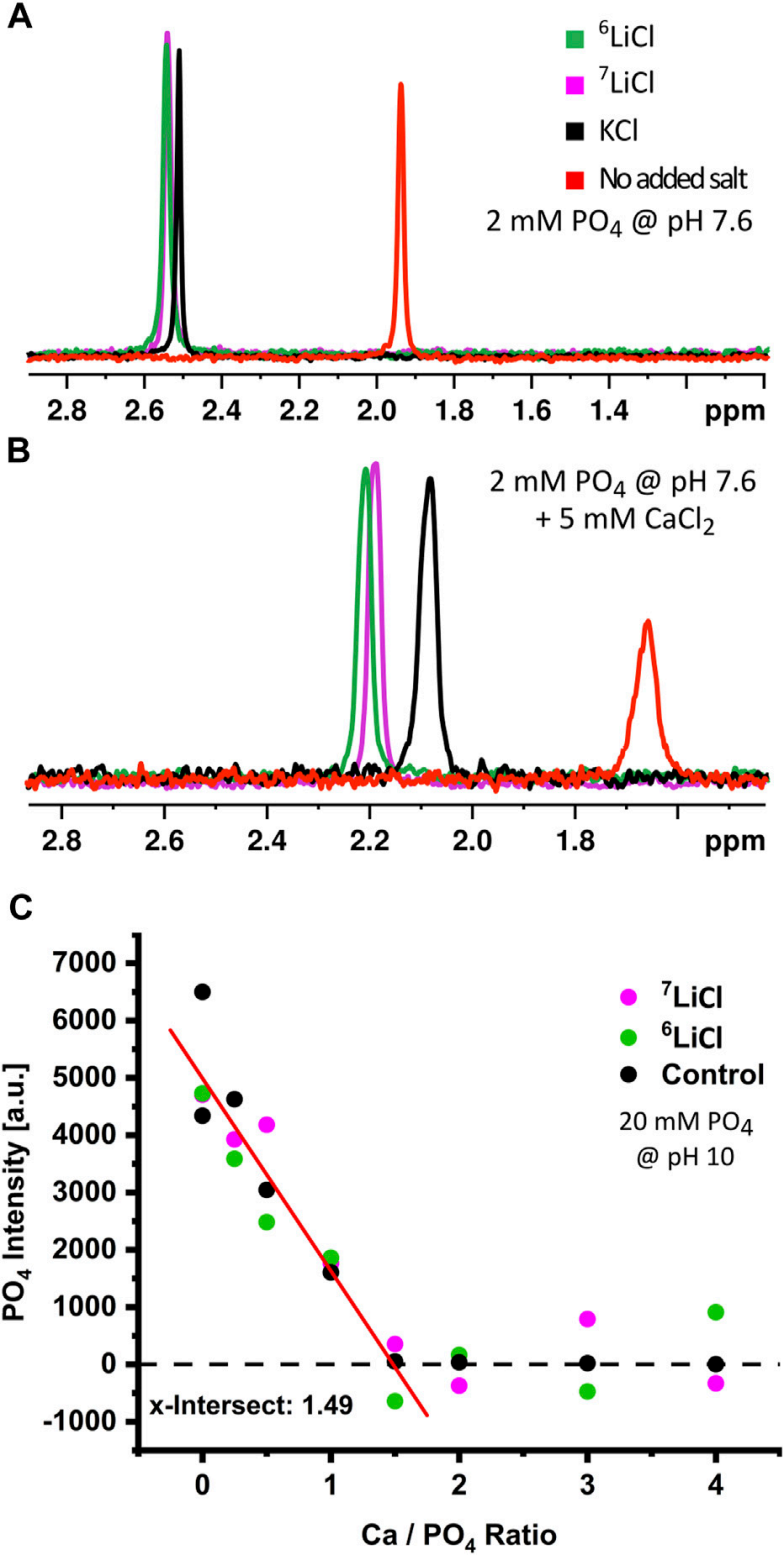
Lithium isotopes incorporate into amorphous calcium phosphate in low quantities. Chemical analysis of ACP in the presence or absence of lithium isotopes. Cation induced chemical shifts of ^31^P in the absence **(A)** or presence **(B)** of calcium at pH 7.6. Phosphate only (red), KCl (black), ^6^LiCl (green), or ^7^LiCl (magenta). **(C)** Stoichiometry determination of ACP in the presence of KCl (black), ^6^LiCl (green), or ^7^LiCl (magenta). The ^31^P-free phosphate signal was monitored during a series of increasing calcium amount with fixed phosphate amount at pH 10. The *x*-axis shows the Ca/PO_4_ ratio after a given calcium addition; the *x*-intercept (1.49) thus indicates the determined Ca:^31^P stoichiometry of formed ACP.

The clear evidence for similar lithium isotope distribution throughout a cell implies direct differential effects of ^6^Li and ^7^Li on ACP within the mitochondrial matrix. We sought to confirm that lithium isotopes incorporate into ACP and to check for any isotopic dependence on lithium interactions with phosphate ions. We conducted ^31^P NMR on solutions of 2 mM monophosphate and 125 mM LiCl at pH 7.6°C and 37°C. We found that the ^31^P NMR-signal line shape and linewidth were almost identical, with a small downfield shift on the order of 0.01 ppm for ^6^Li relative to ^7^Li, as is consistent with a slightly longer bond for the lower mass isotope (Figure 6A). This confirmed that lithium isotopes do not act differentially on phosphate ions prior to the addition of calcium. We then added calcium to these solutions to form amorphous calcium phosphate *in vitro* and measured the ^31^P NMR signal over a period of 5–12 min after calcium was added (Figure 6B). We detected a notable difference in chemical shift between pure ACP and ACP formed in the presence of potassium or isotopic lithium salts, as well as between the monophosphate and ACP lines, confirming that the phosphate is all being complexed into calcium phosphate. This cation-induced effect indicates that ^6^Li, ^7^Li, and potassium all incorporate into ACP structures as they form in solution. Notably, the difference in chemical shift between the ^6^Li-ACP and ^7^Li-ACP is again on the order of 0.01 ppm, which can be attributed to the small mass difference of lithium isotopes and indicates that the lithium is incorporated into ACP in an isotope-independent manner.

Provided the above evidence that lithium isotopes integrate into ACP, we then sought to determine how lithium isotopes alter the stoichiometry of ACP. We utilized a condition of high pH and high ACP precipitation which rendered unbound phosphate detectable and ACP-bound phosphate undetectable, (Supplementary Figure S5). We then compared the change in the inorganic phosphate specific ^31^P NMR-signal upon increasing Ca:PO_4_ ratios. Because the phosphate signal decreases as it is incorporated into ACP with this methodology, an increasing calcium amount in the presence of a fixed amount of phosphate allows the precise titration of calcium to phosphate stoichiometry within the formed ACP. Our analysis identified this Ca:PO_4_ ratio to be 1.49, which agrees with the commonly accepted ACP stoichiometry of 9:6 (Ca:PO_4_) (Posner and Betts, 1975; Wolf et al., 2017), and neither lithium isotope significantly changed overall ACP stoichiometry (Figure 6C). Integration of lithium into the ACP subunit, as previously predicted (Swift et al., 2018), would reduce the observed Ca:PO_4_ ratio. The absence of such a reduction either implies an integration of lithium into the ACP meta-structure outside its core component, e.g., in nanometer scale gaps between Posner clusters of unchanged calcium to phosphate ratio (9:6), or alternatively, a lithium integration rate that is too small to detect with NMR spectroscopy.

In summary, lithium clearly integrates into ACP as it forms, in an isotope-independent manner, and does not alter the global ACP stoichiometry. While the exact location of lithium within the ACP structure remains to be elucidated, its unambiguous presence plausibly affects the characteristics of the resulting ACP and its handling within mitochondria.

### 3.5 Lithium isotopes impart distinct effects on amorphous calcium phosphate aggregation

**FIGURE 7:**
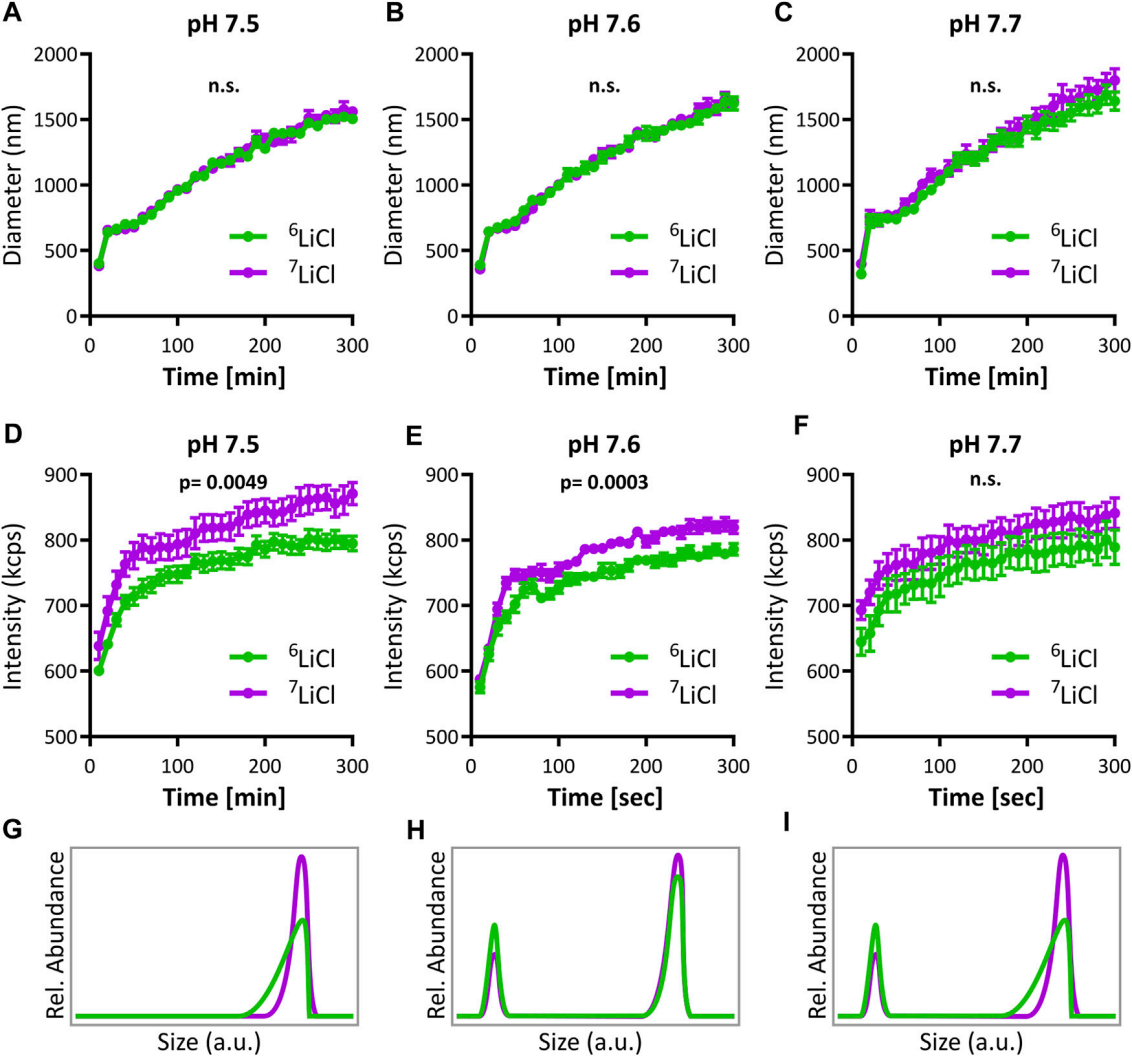
Lithium-6 and -7 differentially promote amorphous calcium phosphate aggregation. Analysis of ACP growth dynamics by dynamic light scattering (DLS). Mean particle diameter of ACP colloids forming in either 125 mM ^6^LiCl (green) or ^7^LiCl (magenta) at **(A)** pH 7.5, **(B)** 7.6, or **(C)** pH 7.7, mean values ± SE, *n* = 10. **(D–F)** Scattering intensity of ACP during the same experiments presented in **(A–C),** respectively, mean values ± SE, *n* = 10. Three conceptual visualizations of ACP particle size distributions plausibly underlying differences in **(D, E)**. In **(G)**, The ^6^Li-ACP and ^7^Li-ACP have the same peak, but the ^6^Li-ACP has a longer tail of smaller particles. In **(H)**, there is a bimodal size distribution, and ^6^Li-ACP has a higher percentage of calcium phosphate particles at the smaller of the two peaks. **(I)** Shows a combination of the cases of **(G)** and **(H)**.

Lithium integrates into ACP, as demonstrated by the above NMR data. At the same time, we found no evidence for differences of spatial distribution related to different lithium isotopes in mitochondria or cells by ICP-MS and NanoSIMS. The different effect of lithium isotopes on mitochondrial calcium handling thus most likely originates in a lithium isotope dependent interaction with phosphate during ACP formation. To explore this, we analyzed ACP formation and aggregation using dynamic light scattering (DLS) in the presence of either ^6^LiCl or ^7^LiCl at a plausible pH range reflecting the mitochondrial matrix environment. Indeed, isotope-specific differences in ACP formation were detected in all three conditions tested. While the average ACP particle size described by DLS was similar (Figures 7A–C), a notable difference in scattering intensities was detected with ^7^Li causing a higher intensity than ^6^Li at pH 7.5 and 7.6, and a similar trend at pH 7.7 (repeated measured ANOVA Figures 7D–F). We applied regression analyses across the last 250 s of relatively stable formation rate that follow the initial burst-like 50 s. Interestingly, the resulting intercepts (but not slopes) of scattering intensity differed between isotopes at each pH tested (all three *p* < 0.0001), indicating a rapid emergence of isotope-specific intensity within the first 50 s that is then maintained. The effect shown here by lithium isotopes differentially acting on ACP formation is plausibly explained by a difference in size distribution amongst the particles. While there are multiple potential size distributions that could explain the observed difference in intensity, Mie scattering theory predicts that a higher intensity corresponds to a higher particle concentration of the largest particles in a given distribution. As the isotope with the highest signal intensity, ^7^Li appeared to promote a higher abundance of the largest ACP granule sizes compared to ^6^Li. Three potential outcomes of this resulting lithium isotope effect on the structuring of ACP have been provided (Figures 7G–I) to allow for conceptual visualization of the DLS results. In any event, ^6^Li and ^7^Li clearly interacted differentially with ACP formation and their presence led to the emergence of ACP with different characteristics. This direct consequence of isotope identity on ACP formation potentially constitutes the main underlying mechanism for lithium isotope specific interaction with mitochondrial calcium storage capacity and stability.

## 4 Discussion

The molecular mechanisms underlying a therapeutic effect of lithium in the treatment of mental health problems remain incompletely understood (Kerr et al., 2018). In this work, we corroborate earlier evidence that lithium interacts with mitochondrial calcium sequestration, a key process during synaptic signal transmission (Shalbuyeva et al., 2007). Importantly, the two naturally occurring lithium isotopes, ^6^Li and ^7^Li, displayed different molecular activities. This primary finding could be the consequence of two different underlying mechanisms: first, an isotope selectivity of lithium membrane transport processes leading to different cellular lithium compartmentalization depending on lithium isotope or, second, an isotope specific interaction of lithium with amorphous calcium phosphate (ACP) in the mitochondrial matrix.

The phenomenon of biological isotopic selectivity has repeatedly been reported (Sherman et al., 1984; Lieberman et al., 1985; Skulan and DePaolo, 1999; Heuser and Eisenhauer, 2010; Balter and Vigier, 2014). The seemingly obvious causative mass difference does not appear to be generally important, because the direction of selectivity does not systematically favor lighter or heavier lithium isotopes (Balter and Vigier, 2014). We applied multiple state-of-the-art mass spectrometry-based methodologies to study lithium isotope distribution across the neuronal plasma membrane, the mitochondrial inner membrane and complete cellular compartmentalization. Respiring mitochondria bathed in an equimolar solution of the lithium isotopes maintained the same isotope ratio as the supporting buffer, in the absence and presence of calcium and over an extended time, when measured by ICP-MS. The same was true for synaptosomes treated and analyzed in the same fashion.

Further, our ICP-MS data indicated no measurable lithium isotope fractionation during lithium uptake by neuronal derived HT22 cells. Although there is no comparable data available in the literature directly relevant to lithium isotopes partitioning across the neuronal cell plasma membrane, it was found that ^6^Li concentrations were increased by 25% compared to ^7^Li in human erythrocytes, suggesting that the erythrocyte membrane is capable of distinguishing between the two lithium isotopes (Hughes and Birch, 1992). It was also reported that neurons in the rat cerebral cortex sustain 50% higher ^6^Li concentrations than ^7^Li when an equal dose of each is administered, possibly due to different uptake efficiencies (Sherman et al., 1984). Contrary to these studies, our findings in HT22 cells correlate well with the absence of differential distributions of lithium isotopes across mitochondrial and synaptic membranes, as well as the lack of spatial distribution differences of lithium isotopes within NIH/3T3 cells. Applying the high spatial and signal resolution of NanoSIMS to lithium treated cells produced clear lithium isotope signals, resulting from their distribution throughout the cell as well as certain cellular topological effects, yet no disparity in isotope distribution was apparent to any degree, anywhere within the cell or across its plasma membrane. While we cannot rule out a lithium isotope selective distribution in any living system, we are confident it did not play a role in the experimental model systems employed in this study.

The lack of differences in isotopic lithium distribution across mitochondrial membranes suggest that an alternative mechanism is involved in lithium isotope selective effects that act on mitochondrial calcium handling. Such mechanisms may operate through amorphous calcium phosphate clusters (ACP). Mitochondria feature the ability to transiently store massive amounts of calcium in the form of osmotically inactive, gel-like ACP (Rossi et al., 2019). Major gaps remain in our understanding of this critical process, namely, how ACP is formed, maintained, utilized and dissolved in the matrix. Here, we show specific interactions of lithium isotopes with mitochondrial ACP formation resulting in altered MPT onset and calcium accumulation. This was supported by ruling out lithium effects on mitochondrial respiration (Figure 1D) and calcium efflux rate (Figures 2C, F), as well as effects on non-MCU calcium accumulation and resting membrane potential (Supplementary Figure S3). One remaining plausible mechanism is the interaction of lithium with the established drop in mitochondrial membrane potential during calcium uptake through the MCU (Vergun et al., 2003; Vergun and Reynolds, 2004; Komary et al., 2010). Such effects may influence the dynamic shaping of ACP. In support of molecular agents such as lithium influencing ACP formation directly, we previously identified that ATP alters ACP density and mitochondrial calcium capacity (Deline et al., 2021). This biological outcome is well in line with the ability of magnesium-complexed ATP to delay the crystallization of ACP to hydroxyapatite in an abiotic system, from the order of hours to days (Blumenthal et al., 1977). Likewise, citrate and phosphocitrate delay ACP crystallization (Tew et al., 1981; 1980).

The constituent smallest structural unit of ACP is generally assumed to be a highly symmetrical sphere about 1 nm in diameter and in a Ca_9_(PO_4_)_6_ stoichiometry (i.e., the Ca:PO_4_ ratio), often referred to as a “Posner” cluster (Lehninger, 1970; Posner and Betts, 1975; Wolf et al., 2017; Strubbe-Rivera et al., 2021). Substitution of the central calcium by two lithium ions was calculated to be an energetically favorable structure, whereas substitution at this site with other cations, e.g., lead, would destabilize it (Swift et al., 2018). In our experiments, we indeed demonstrated incorporation of lithium into ACP, but were unable to detect a shift in the global stoichiometry as expected by replacing a calcium by two lithium ions. Either the fraction of lithium containing Posner clusters was too small to detect or lithium is integrated in between Posner clusters of unchanged stoichiometry during its aggregation into higher order structures. Either way, the isotopic identity of lithium led to specific consequences in the higher order particle size distribution. This observation on the abiotic level is a putative origin of altered mitochondrial ACP structure and in turn isotope specific changes in calcium capacity and MPT propensity.

Our observations of lithium isotope specific effects on ACP formation and mitochondrial calcium handling greatly expand on the little explored area of mitochondrial targeted lithium bioactivities (Shalbuyeva et al., 2007), and provide plausible mechanistic insight into the previously reported lithium isotope dependent effects on animal behavior (Ettenberg et al., 2020). Calcium is a well-known signal of neurotransmitter release and a myriad of other cellular functions (Islam, 2020). Mitochondria are undisputed regulators of cytosolic calcium concentration, mostly by rapid calcium uptake and clearance following a calcium spike (Nicholls, 2005). Indeed, calcium dysregulation and mitochondrial dysfunction are often associated with neuronal disorder (Khacho et al., 2019; Calvo-Rodriguez et al., 2020; Cascella and Cecchi, 2021). The exact role and contribution of mitochondrial ACP to cellular calcium signaling and homeostasis, even if apparently straightforward, has yet to be fully elucidated. It is, however, the crucial link between our observations and phenotypic manifestations.

Importantly, we found that low lithium concentrations, as clinically maintained, are sufficient to affect liver mitochondrial calcium handling in an isotope specific manner. These are primarily not affecting mitochondrial calcium uptake but are instead explained by a differential influence on the organization and/or stability of mitochondrial ACP, since the higher calcium accumulating condition (^6^LiCl) is also the least effective at triggering calcium induced MPT. We took multiple precautions to reduce any possible difference in calcium contamination amongst the conditions tested: i) each condition was formulated by adding a working salt stock to a common calcium assay buffer master mix, ii) the calcium assay buffer master mix was stripped of calcium using the resin Chelex-100 (potassium form), and iii) technical replicates of each condition were randomized on multi-well plates.

We observed altered calcium handling in brain mitochondria only at higher lithium concentrations. However, clinical lithium concentrations in plasma may not reflect intra-neuronal concentrations very well. Such possible discrepancies may arise through the dramatic fluctuations in neuronal membrane potential, which have been modelled to drive lithium accumulation to an estimated concentration of 8 mM (Kabakov et al., 1998). In any case, the diametrically opposite effect of lithium on mitochondria of different tissue origin is clear evidence for a very specific interaction with diverse mitochondrial calcium handling mechanisms. A tissue difference in mitochondrial calcium use and capacity is well documented, including stark differences between brain and liver mitochondria (Chalmers and Nicholls, 2003). In fact, we ourselves were unable to provoke a calcium induced MPT in brain mitochondria, which readily respired unhindered at a calcium load fatal for liver mitochondria. Under such conditions of extreme matrix ACP abundance, an altered higher order structure brought about by the presence of lithium conceivably manifests in a brain-mitochondria specific manner.

Tissue-specific ordering of ACP may also be a function of lithium availability. The best-known pathway of mitochondrial lithium accumulation is through the sodium/calcium/lithium exchanger (NCLX), which does exhibit tissue-specific activity levels (Rysted et al., 2021). Brain and liver mitochondria, synaptosomes and cultured cell were not selective towards specific lithium isotopes in our hands, yet future work is required to understand if total accumulated lithium is different in a tissue specific manner. Further, the expression of molecular components driving mitochondrial calcium and phosphate accumulation may adapt during extended lithium treatment of cells or organisms. Our conclusions derive from experiments with isolated mitochondria unaffected by such considerations, but these will have to be addressed in further translational work.

What remains enigmatic is the underlying characteristic of lithium isotopes to differentially interact with ACP formation. Both ^6^Li and ^7^Li share a highly similar electron shell and are thus thought to interact with their environment in an alike fashion (Renshaw, 1987). There is also little explanatory potential in the mass difference between lithium isotopes, which does not appear to be important in lithium isotope distribution in mammalian organs (Renshaw, 1987; Balter and Vigier, 2014). By exclusion, this leaves nuclear spin properties as one major remaining difference, with ^6^Li having spin-1 and ^7^Li spin-3/2. This difference in nuclear spin results in significantly different gyromagnetic and quadrupolar moments for the two isotopes, which in turn dictate how strongly these spins interact with their environments. Indeed, the building block of ACP, the Posner cluster, has been modelled to feature extraordinary quantum properties relating to nuclear spin, i.e. ^31^P spin coherence times on the order of several hours, and a symmetry-based bonding rule, known as quantum dynamical selection (Fisher and Radzihovsky, 2018; Swift et al., 2018). The presence of lithium within Posner clusters may result in a spin-related interaction between lithium and phosphorus, which could impact bonding rates via the decoherence of the relative phases between phosphorus nuclear spin within the framework of quantum dynamical selection (Fisher, 2015; Swift et al., 2018).

Apart from the fascinating question of underlying physical mechanisms, a putative differential clinical efficacy of ^6^Li versus ^7^Li in the treatment of bipolar and other mental health disorders should be investigated to benefit future therapeutic formulations. While beyond the scope of this study, the distinct isotope specific effects of lithium on liver and brain mitochondria clearly warrants attention in future clinical trials.

## Data Availability

The original contributions presented in the study are included in the article/Supplementary Material, further inquiries can be directed to the corresponding author.
