# Supplementary material for: Lithium isotopes differentially modify mitochondrial amorphous calcium phosphate cluster size distribution and calcium capacity
Source: Front Physiol. 2023 Sep 15;14:1200119. doi: 10.3389/fphys.2023.1200119 (PMC10540846; doi:10.3389/fphys.2023.1200119)
Supplement: Supplementary file 1 [file Image1.pdf]

frontiers

Supplementary Material

Lithium isotopes differentially modify mitochondrial amorphous calcium phosphate cluster size distribution and calcium capacity

Marshall L. Deline[1], Joshua Straub[2], Manisha Patel[2], Pratigya Subba[1], Martin Grashei[3], Frits H.A. van Heijster[3], Philip Pirkwieser[4], Veronika Somoza[4,5], James D Livingstone[6], Michael Beazely[6], Brian Kendall[7], Michel J.P. Gingras[8,9], Zoya Leonenko[8,10], Carmen Hoeschen[11], Gertraud Harrington[11], Katharina Kuellmer[1], Wangqing Bian[1], Franz Schilling[3], Matthew P.A. Fisher[2], Matthew E. Helgeson[12], Tobias Fromme[1,13]*

*** Correspondence:** Corresponding Author: fromme@tum.de

1 Supplementary Figures

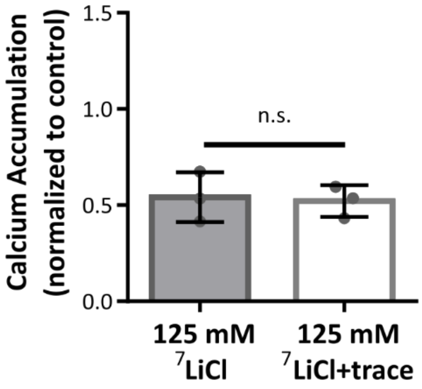

Supplementary Figure 1. Lithium isotope effect on liver mitochondrial calcium accumulation is not the result of trace ion composition. A higher relative abundance of Na^+, Mg^{2+}, and Cu^{2+} was found in the ^{6}LiCl stock compared to the ^{7}LiCl. Maximal calcium accumulation of mitochondria in 125 mM ^{7}LiCl was unaffected by the increase of these trace ions when incorporating ^{6}LiCl trace abundances (^{7}LiCl+trace, open bar) when compared to those treated with an unaltered stock (^{7}LiCl, solid bar). Means of technical replicates are plotted, n=3.

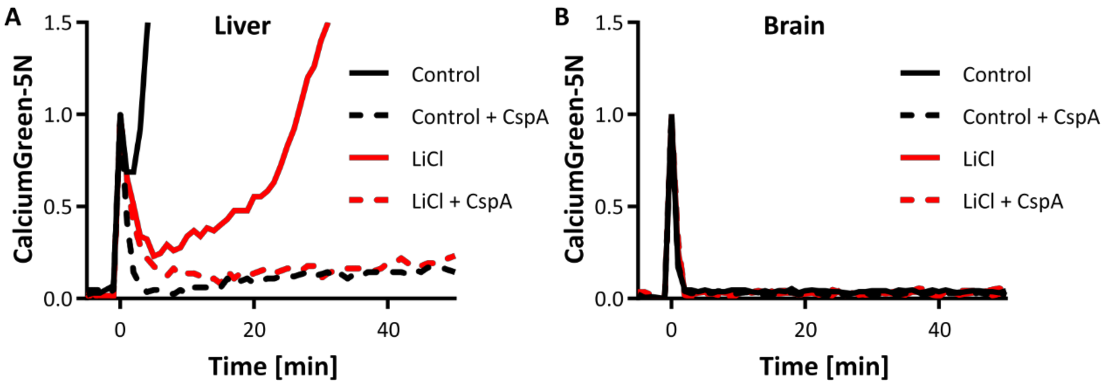

Supplementary Figure 2. Calcium efflux following large calcium bolus addition to liver mitochondria is the result of MPT, while calcium efflux does not occur in brain mitochondria when treated in the same manner. (**A**) Representative trace from 3 biological replicates of liver mitochondria suspended in calcium assay buffer supplemented with 125 mM KCl (Control, black) or with 125 mM LiCl (red), in the presence (dashed line) or absence (solid line) of the MPT inhibitor 5 µM Cyclosporin A (CspA), and treated with a 350 nmol/mg calcium bolus at time 0. (**B**) Representative trace from 3 biological replicates of brain mitochondria suspended calcium assay buffer supplemented with 125 mM KCl (Control, black) or with 125 mM LiCl (red), in the presence (dashed line) or absence (solid line) of 5 µM CspA, and treated with a 350 nmol/mg calcium bolus at time 0.

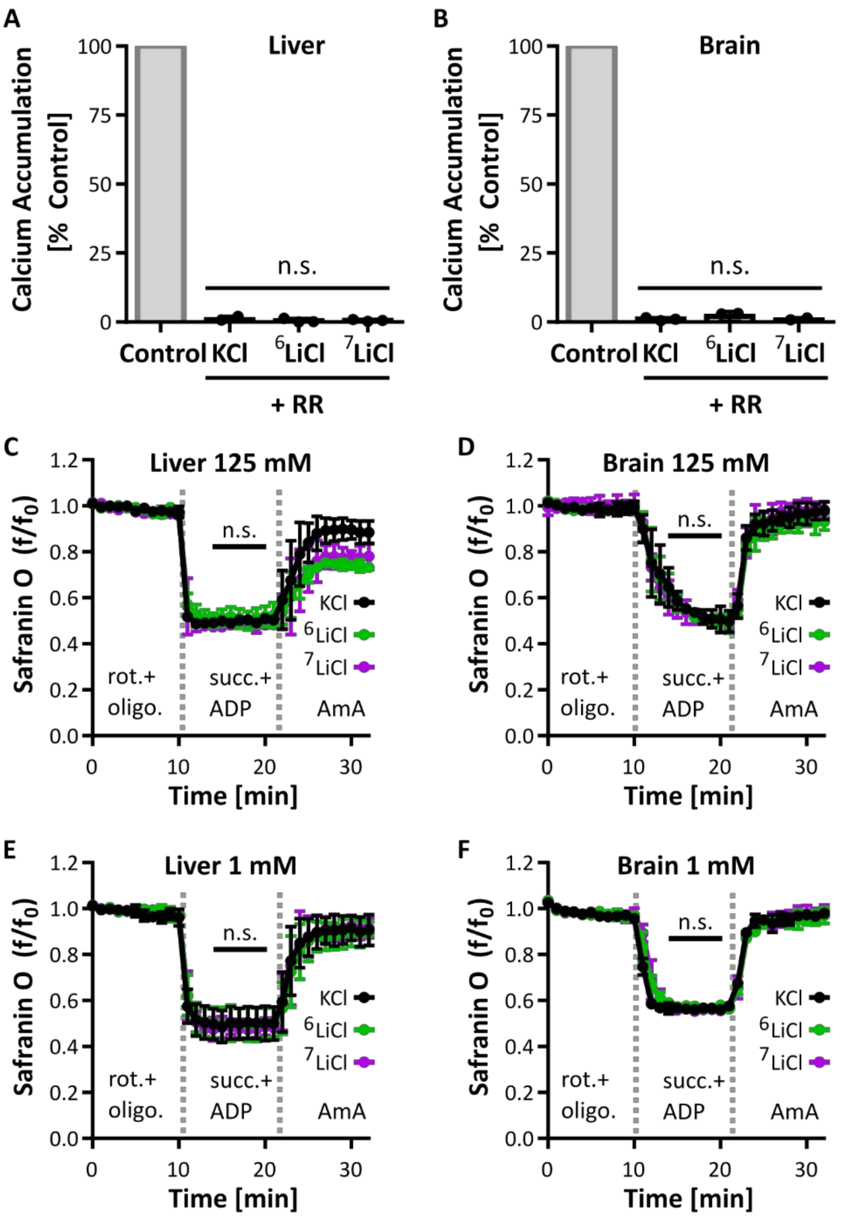

Supplementary Figure 3. Calcium uptake is specific to the mitochondrial calcium uniporter in the presence of lithium. (**A**) Brain or (**B**) liver mitochondria were suspended in Calcium Assay Buffer supplemented with 0.5 µM ruthenium red (RR) and either 125 mM KCl (Control), 125 mM ^{6}LiCl, or 125 mM ^{7}LiCl. Calcium accumulation was determined as in Figure 2 and is plotted as percent of the calcium accumulation by Control buffered mitochondria in the absence of RR. No statistical significance was identified when comparing the ruthenium red treatments by ANOVA, n=3. The resting membrane potential of both liver and brain mitochondria is unaffected by the presence of lithium. The membrane potential of mitochondria buffered by 10 mM HEPES supplemented with rotenone (rot.), oligomycin (oligo.), and either KCl (black), ^{6}LiCl (green), or ^{7}LiCl (magenta) were assessed by following changes in the fluorescence of 2 µM Safranin O with a TECAN microplate reader when 2 mM succinate (succ.) and 1 mM ADP was applied. The contribution of the membrane potential to the fluorescence change was confirmed by applying 2.5 µM of the complex III inhibitor, antimycin A (AmA). The average change in fluorescence for a 5 min window following addition of

succinate and ADP was compared for **(C)** liver or **(D)** brain mitochondria supplemented with 125 mM KCl, ^{6}LiCl, or ^{7}LiCl. No difference was found in any condition when assessed by one-way ANOVA, liver n= 4, brain n= 3. The average change in fluorescence for a 5 min window following addition of succinate and ADP was compared for **(E)** liver or **(F)** brain mitochondria supplemented with 124 mM KCl and either 1 mM ^{6}LiCl or ^{7}LiCl. No difference was found in any condition when assessed by one-way ANOVA, liver n=4, brain n= 3.

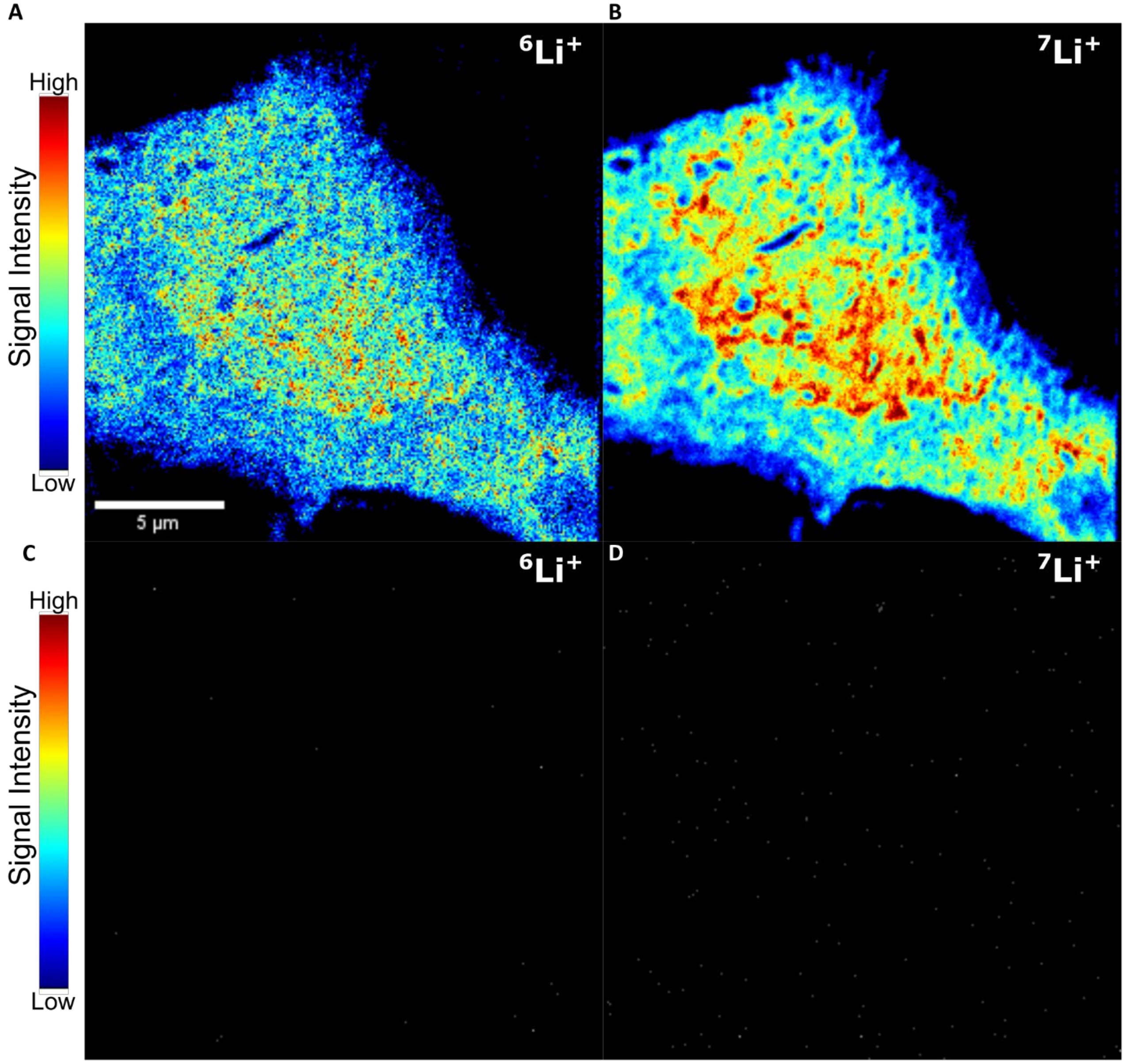

Supplementary Figure 4. (A-B) Lithium isotope signals from an NIH/3T3 cell treated with 400 µM LiCl for 48 hrs, as measured by NanoSIMS and shown in Figure 5. (C-D) Lithium isotope signals from an NIH/3T3 cell treated with 400 µM NaCl for 48 hrs, as measured by NanoSIMS.

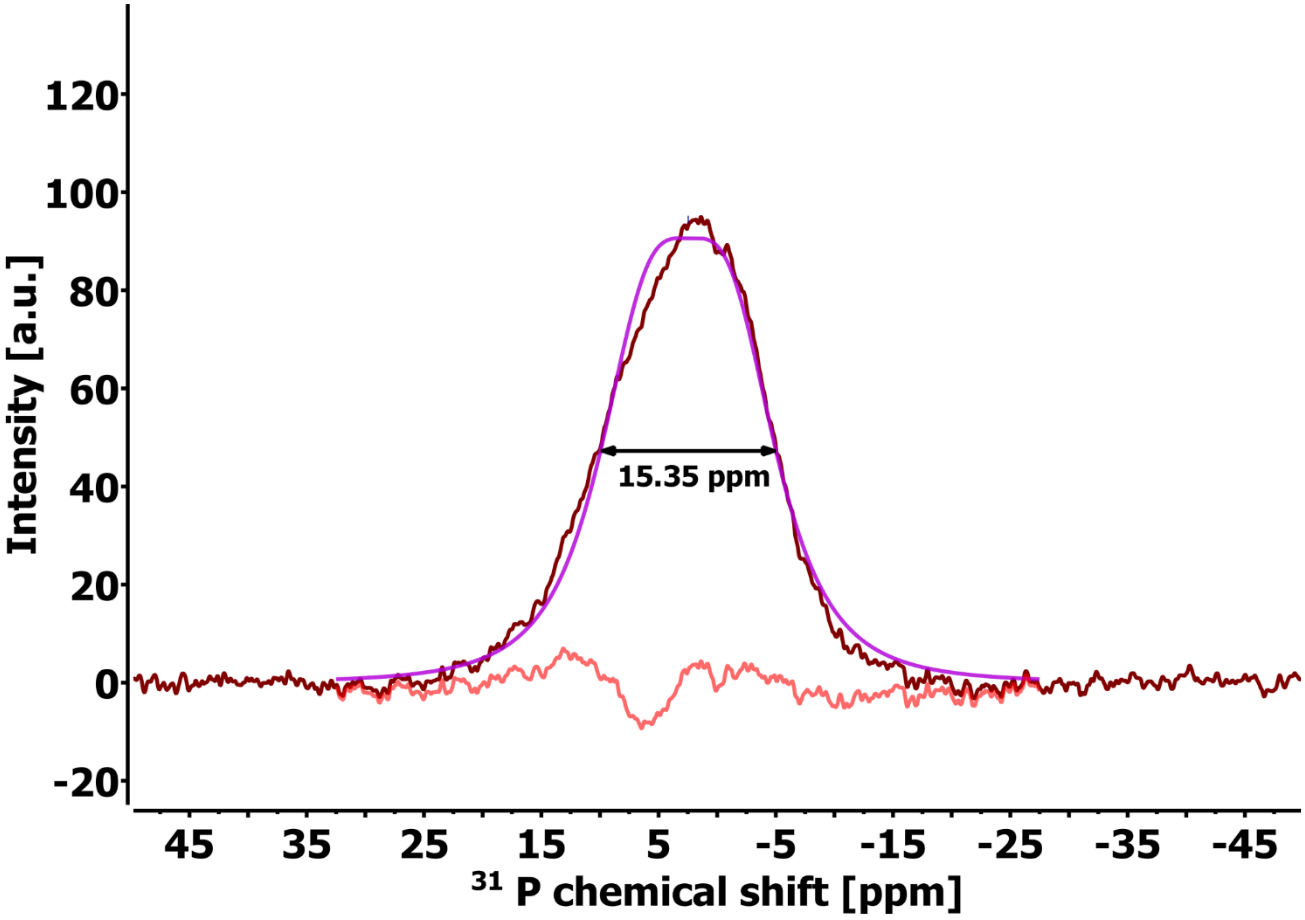

Supplementary Figure 5. Bulk ACP generates a broad line ^{31}P NMR spectrum. Phosphate bound within ACP generates a broad ^{31}P NMR signal (64 averages, SNR 40) of low SNR, rendering it essentially undetectable in the experimental settings which were to determine ACP stoichiometry (only 1 average, SNR 94 - 347).
